# Blogging as a Viable Research Methodology for Young People With Arthritis: A Qualitative Study

**DOI:** 10.2196/jmir.3608

**Published:** 2015-03-05

**Authors:** Julie Prescott, Nicola J Gray, Felicity J Smith, Janet E McDonagh

**Affiliations:** ^1^University of BoltonDepartment of Education and PsychologyBoltonUnited Kingdom; ^2^Green Line Consulting LtdManchesterUnited Kingdom; ^3^UCLSchool of PharmacyLondonUnited Kingdom; ^4^University of ManchesterCentre for Musculoskeletal ResearchManchesterUnited Kingdom

**Keywords:** young people, blogging, qualitative research, arthritis

## Abstract

**Background:**

The development of services that are responsive to the needs of users is a health policy priority. Finding ways of engaging young people in research to gain insights into their particular experiences, perspectives, and needs is vital but challenging. These data are critical to improving services in ways that meet the needs of young people.

**Objective:**

Our aim was to evaluate Web-based blogging as a viable method for understanding the daily experiences and condition management strategies of young people with juvenile arthritis.

**Methods:**

To meet the objectives of the study, a qualitative approach was required to gather information on the experiences and perspectives of young people regarding the management of their condition and its daily impact. In collaboration with a group of young people with arthritis, a custom website was developed. This website provided the opportunity for young people (aged 11-19) with arthritis from a United Kingdom pediatric hospital to contribute blogs. It was designed so that young people were free to write about whatever was important to them, but the site also included some structure and prompts to facilitate the writing of blogs. Qualitative analytical procedures were employed, supported by NVivo software.

**Results:**

Engagement in the study by young people was variable in terms of their participation rates, frequency of website visits, and the length of their blogs. Young people used the site in different ways, some responding to the website categories and prompts that the team created, while others used it as a diary to record their experiences and thoughts. In line with principles of qualitative inquiry, the data collection was participant-led. Young people were in control of what, how much, and how often they wrote. However, some young people expressed difficulty regarding knowing what they should blog about. For a number of reasons, discussed here, the blogs may also not be fully reflective of experiences and perspectives of the participants. However, the data obtained provided insights into young people’s experiences of living with arthritis and their use of medicines in the context of their daily lives.

**Conclusions:**

Web-based research with young people presents opportunities and challenges for researchers. Web-based blogging methodology has the potential to give young people and parents the space and empowerment to express their own ideas and concerns. However, this project suggests that it might not be the best way to engage a large diverse group of young people and might most effectively be combined with other approaches. Despite these limitations, the study provided valuable data about the experience and impact of living with a long-term condition from the perspectives of young people with arthritis.

## Introduction

In 2003, Internet-based research was viewed as still being in its infancy [1]. More than 10 years on, the Internet is widely employed in many aspects of daily life and is increasingly being used within academic research. In particular, Web-based surveys have become very popular [2,3]. They have been noted for their potential to reach very large audiences, inexpensively, with rapid replies [4,5]. Although for some research, they may be limited by anticipated response rates; in other studies with specified populations, and with the use of more sophisticated software, they can be valuable [6,7].

The potential value of Web-based research for qualitative studies is less clear. The Internet has been used for a diverse range of studies using traditional qualitative data-gathering techniques such as interviews [8,9] and focus group research [10]. Experience is considerably more limited regarding the potential use of blogs [11,12]. A blog (short for weblog) has been described as a Web-based diary in which the blogger can freely record opinions and recount experiences. Blogs can be produced by single or multiple authors, and they provide information to tell a story, and/or represent particular experiences or perspectives on any issue [13]. Bloggers are often part of a blogging community that centers on a common interest (eg, Olive’s 2013 study on surfers [12]). By their very nature, it seems that blogs may provide an excellent research opportunity; however, they are currently underutilized in social science and health services research. Hookway [11] compares blogs to offline diary research, suggesting blogs allow for more extensive research opportunities through the potential anonymity of blogs (both the anonymity of the blogger and those that comment on other blogs) and the ability to enable researchers to reach a wider audience. Diary research has been used to investigate, among other issues, health behavior [14-16]. Benefits include the ability to collect sensitive information [14] and the ability to capture an “everchanging event” [14], where the time between an event and its recording is reduced, resulting in less memory impairment and reduced reconstruction of the event compared to other qualitative methods such as interviews and focus groups [17]. Olive [12] found blogging a good means of data collection since there were no location constraints to blogging—as long as you had access to a computer and the Internet—and the process of blogging could also allow the blogger to be reflective.

Web-based resources for gaining information and sharing experiences on medical/health issues are increasingly popular [18], and these too may provide data for research, especially for populations with specific medical/health issues [18]. Malik and Coulson [19] used Web-based methodologies by examining a password-protected Web-based support group to understand the lives and experiences of young people with inflammatory bowel disease (IBD). A particular benefit of Web-based support groups, as featured in Malik and Coulson’s [19] study, is that the groups are available 24/7, which enables people to receive support and advice when more traditional resources are unavailable. Web-based support groups also offer people the convenience of support with similar people from within their own homes [20], which may be an important feature for people with long-term conditions who may have difficulty attending face-to-face meetings. Web-based groups also offer anonymity, which may increase self-disclosure [20,21]. Another study [16] of 6 young people aged between 11 and 16 years with IBD used an audio (offline) diary methodology. Researchers claimed that the diary methodology was a viable method for communicating and engaging with young people. Participants were able to record what they wanted, when they wanted, with researchers able to gain an understanding of the lives of a diverse group of young people.

With all Web-based methodologies, researchers must be aware of their sample: their sample’s ability to blog, having an interest in blogging, and access to the resources. However, research suggests that many young people aged between 13-19 blog [22,23]. Indeed, Hookway [11] posits that bloggers need low technical competence in order to blog, and therefore it is a methodology that is potentially inclusive. In the United Kingdom, however, inequalities in relation to access and use of the Internet have been identified [24,25] with boys, older children, and middle class children more likely to have access. This suggests there is still a digital divide for young people that should be borne in mind when considering Web-based research with specific groups. Despite inequalities in access, previous studies in the United States [23] and United Kingdom [26] on gender differences in blogging have suggested that females aged 15-17 blog more than their male counterparts. Gender differences have been found to exist in how and why people blog [26,27] and with female-authored blogs being commented on, and noted less [26].

The development of services that are responsive to the needs of users is a health policy priority in the United Kingdom, and the value of qualitative methods in enabling researchers and policy makers to gain insights into the experiences and priorities from the perspective of service users is recognized. In particular, research into the perspectives of young people that can inform service development is very limited [28]. It is recognized, however, that young people with long-term conditions have very distinct needs and concerns in their daily management and preferences regarding service provision and delivery. Finding ways of engaging young people in research—to gain insights into their experiences, needs, and priorities—is vital but challenging [28].

This paper describes Web-based blogging as a method for understanding the daily experiences and condition management of young people with juvenile arthritis. A systematic review of qualitative studies involving the experience of children and young people living with juvenile idiopathic arthritis (JIA) has been conducted [29]. None of the resulting 27 studies, completed up to 2011, involved Web-based blog data, although there were a small number involving video diaries [29]. The overall aim of the research was to investigate the relationships between identity and medication use among adolescents with arthritis and to explore the role of health professionals in delivering services to this group. This paper discusses how using a qualitative Web-based bespoke blogging website, shaped by young people, enabled the research team to meet the overall research objectives and engage with young people with a long-term health condition.

## Methods

### Study Setting and Participants

Young people (aged 11-19 years) with arthritis who attended a clinic at a major UK pediatric hospital were invited to take part in the study. The clinic served a diverse group of young people from the West Midlands area.

### Ethical and Research Approvals

Approval for the study was given by Coventry & Warwickshire Research Ethics Committee in July 2012 (ref 12/WM/0184) and the Hospital Directorate for Research and Development*.*

### Procedure

Young people and their parents were identified from the patient list of a rheumatology clinic in a large UK pediatric hospital. Eligibility criteria for young people required that they had confirmed child/adolescent onset arthritis, were aged 11-19 years, and had no cognitive/learning disorder that would prevent their ability to provide data. All eligible young people (and their parents) were invited to participate by letter (n=107) and followed up during routine clinic appointments (n=70). Young people under the age of 16 gave their own assent to take part, coupled with a parent’s consent. Young people aged 16 and over provided their own consent. Consenting participants were given an information pack about the “Arthriting” website and a personal login code. To maximize relevance and uptake, development of the website was undertaken in partnership with a young people’s user group and included stringent security processes to ensure data protection and participant safety (eg, password-protected logins, secure firewalls, regular moderation). The website was also functional on smartphones, recognizing that many young people (and parents) access the Internet this way. Once registered, participants were asked to choose a nickname and password for future logins. On completion of the 2-month period, participants were sent a letter and online shopping voucher to thank them for their time.

### Website Design

**Figure 1:**
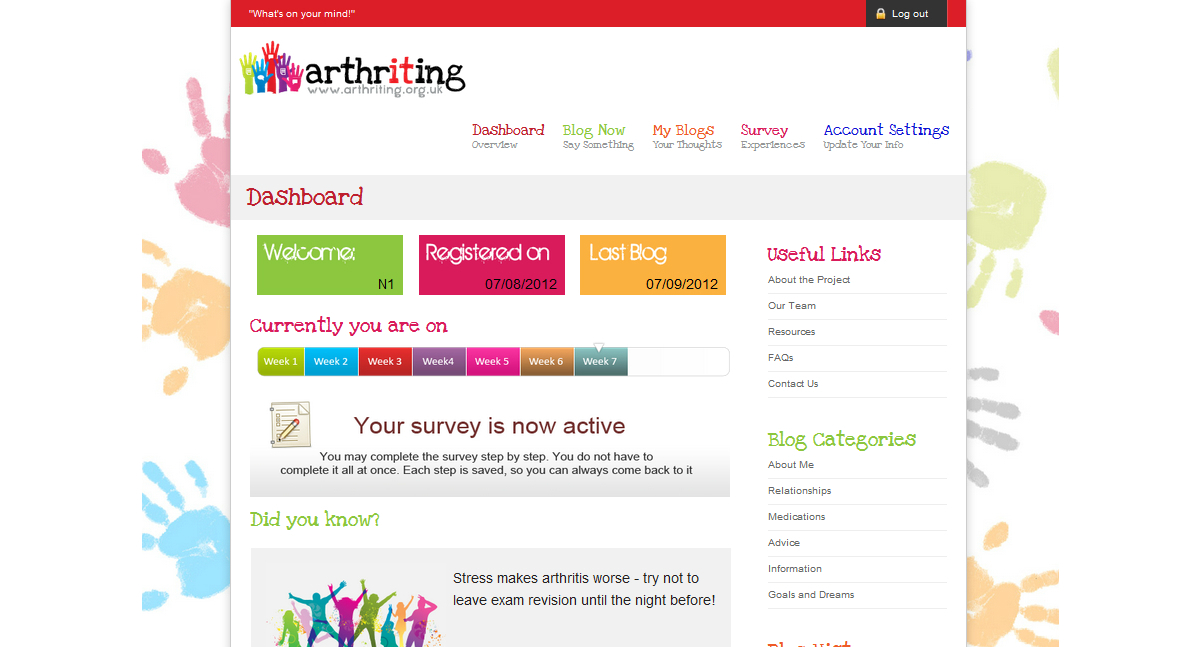
Arthriting website logged-in dashboard (dummy demo testing user).

The young people contributed to the design of the website by attending a discussion with the website builders and members of the research team. Many of its features were decided in partnership with the young people, who had strong ideas regarding the look and operation of the website. One young person suggested the name “Arthriting” and the logo for the project and website. The website had a secure login function for young people to contribute blogs. Once logged in, participants had a project “Dashboard”, which showed them where they were in the project and gave them a launching-off point to the blog space (see Figure 1). To ensure confidentiality of data, blogging could not take place as a community, but privately as individuals. Some of the young people expressed a wish to communicate with each other, but due to the potentially sensitive nature of the material and desire of others that blogs were private, a decision was made to have a closed individual blog rather than an open group blog method. Thus, blogs were not open for comment by others.

### Blog Structure

To achieve the objectives of the project, the research team generated an initial list of blog categories to give young people ideas for the topics and types of messages that they might post (eg, thoughts about identity, the arthritis condition, medication, and the use of health services). These “blog categories” were developed and refined in discussion with the young people.

When using the blogs, participants were able to choose their own font style and color and choose an emoticon to express how they were feeling that day. They were invited to choose a blog category and to write their own title for the blog. They could also indicate their feelings in a “smiley status”. Young people could edit or delete blogs at any time during their 2-month engagement with the project. There were no minimum or maximum blog lengths or number of blog entries that any person could post. We hoped that they would visit and revisit during their 2-month stay.

### Data Collection

The young people’s information sheet gave the following guidance about blogging:

You will then be invited to write down online what you think of your medicines (both injectable ones as well as those you take by mouth), and how medicines fit—or indeed do not fit—into your everyday lives. It will be like writing a diary but online and like a diary, entries do not have to be every day nor do they have to be lengthy—only just enough to give others a picture of what life is like for you with your arthritis and your medicines. You can take up to 2 months to cover all the issues that you want to tell us about.

Basic demographic and relevant clinical data (which we asked permission to extract during the consent process) were recorded. These data were age, sex, ethnic group, age at diagnosis, and number of medications.

### Data Processing and Analysis

For analysis of the blog data to meet the objectives of this paper, a subgroup of the wider multidisciplinary project team (the authors of this paper) first examined each individual blog to provide a case-by-case overview of the themes of each individual’s entry. From this, a coding frame was developed. As analysis proceeded, this was modified and refined using constant comparison techniques, in which all items of data assigned a particular code were appraised for similarities and divergences from those already coded. To ensure the reliability of analytical procedures, all stages of the data processing, coding, and analysis involved at least 2 members of the research team. Computer software (NVivo) was used to assist in the data management and handling.

## Results

### Overview

In total, 107 young people were eligible and invited to take part, of which 36 young people completed consent procedures and 25 registered with the site. At least one blog was received from each of 21 young people. In addition, 6 parents also registered and completed at least one blog. The sample of the 21 young bloggers broadly reflected the wider clinic population in terms of age and type of arthritis diagnosis, but white young people and females were overrepresented in our blog sample. This may be viewed as consistent with literature showing that female adolescents are more likely to use online health information sources [25]. Table 1 shows characteristics of the 21 young people who contributed at least one blog.

We asked each young person and parent to choose a nickname for their blog and to avoid linking it closely to their real name. The moderator did not know the identity of the bloggers, but the clinical staff did. Where the clinical staff determined that the nickname was too close to the blogger’s real name, we retained the first and last letter and put two full stops “..” in between, regardless of how many other letters there actually were. Blogs have not been edited by the team, but some examples are extracts from a longer blog.

**Table 1:** Characteristics of participants.

Characteristics		n
**Age at recruitment**		
	11-15	18
	16-19	3
**Gender**		
	Female	17
	Male	4
**Ethnic group**		
	White	17
	Non-white	4
**Age at diagnosis**		
	<11 years	13
	11+ years	8
**Time since diagnosis**		
	1-5 years	8
	6-10 years	4
	11+ years	8
	Not known	1
**Type of juvenile arthritis**		
	JIA	19
	Other	2

### Young People’s Approach to the Site

The following quote illustrates a young person’s understanding of the study and their role in it: “Taking medication every day is hard, and living with the pain of arthritis is harder, and over the next few weeks, I hope to blog and give you a better understanding of what it’s like ‘living with arthritis’ and ‘being me’.” [BeanyBabe96, female, age 16].

There was great variability in the frequency of visits to the site by young people and in the length of blogs, with some young people writing frequently and at length and others writing one or two blogs over the duration of the project. Among the young people who blogged, the average number of blogs posted was 8, and the range 1-36. Among the 6 parents, the average number of blogs posted was 4, and the range 1-12. In total, 187 blog entries were contributed.

The young people approached the site and blogging in different ways. Some were guided by the “blog categories” listed on the site. They worked through these, contributing thoughts and experiences that they felt were relevant. This could provide a young person’s perspectives on a broad range of issues. For example, the following were contributed by a female blogger, aged 13, nickname 123456:

Hi today I’m going to talk about medication and how I feel about it. Honestly it doesn’t really affect me most of the time); it doesn’t make me feel different from anyone. I’m glad I take medication because without it I wouldn’t be well. However sometimes when I am angry I don’t feel like taking it because I can’t be bothered and think why should I have to take it. WHY ME????

Hey, so my dreams and goals. Well, when I’m older I want to become a doctor. I’ve always wanted to become a doctor even from a young age, but after I got diagnosed I saw how much the doctors helped and cared for me. I would like to do that for some to. My illness won’t affect my future a lot; hopefully it stays in control and then everything will be alright.

So relationships...it doesn’t really affect me at all I’m still the same person I was before I got diagnosed. My relationships with other are the same, I’m a bit more moody than I used to be which sometimes makes relationships with others hard because I just want to be by myself, but other than that it doesn’t affect me at all!!!’

Other bloggers adopted a more diary-like approach recording their experiences, views, and perspectives with no particular reference to the blog categories. These blogs could provide insights in the context of current priorities and everyday concerns of young people. Thus the blogs may not necessarily explicitly focus on the experience of arthritis and medicines but still do demonstrate how these issues relate to, or impact on, wider aspects of a young person’s life. The blog of “flower123”, a 14-year-old female blogger, is a fascinating example of this approach. A typical entry recounted experiences at school and social activities with family and friends:

Yesterday after school I went to my dance class I had loads of fun !! Today I Finally got my guitar tuned YAY !! I can now hopefully try to teach myself how to play also me and my friend [girl’s name] were on Skype playing our guitars (really badly :/) !!!!!

Although flower123’s blogs do not always focus on arthritis and medicines, those that do provide genuine insights into the place, priority, and impact of arthritis and medicines for the young person. There is a series of references to “my injection”, identified by the drug name in her very first entry as Enbrel (the brand name for the biological medicine etanercept) but never referred to by name again. These events seemed to have a profound effect on her. We present them here with the blog dates for reference:

28thSeptember 2012—Feel rubbish today :( got a cold. Had my injection last night didn’t hurt at all !! YAY !!

2ndOctober 2012—yesterday I had my first injection of the week and it didn’t hurt !! YAY :)

10thOctober 2012—on Monday I had my injection stung a bit not as bad as it has done but it’s OK now

17thOctober 2012—on Monday had my injection it didn’t hurt but it was uncomfortable through my leg I could feel it spreading throughout :/ 

23rdOctober 2012—yesterday I had my injection and it didn’t hurt a bit YAY !!!!

20thNovember 2012—yesterday when I had my injection it hurt a bit :/ it hurt when it was going into my leg :(

23rdNovember 2012—but I feel better today still not 100% and I didn’t go to school today :/ x had my injection yesterday when I was ill and it hurt :

flower123, female

After writing weekly about the first few injections, there was a gap of almost a month before the next blog. Perhaps the intervening weeks had been uneventful and straightforward, as the post-gap entries describe more painful experiences. The blog ended on November 29, so it is unclear whether the situation improved. The blogs of flower123 show that her main priorities were activities with her friends and family; her arthritis seemed not to restrict her. The injections were sometimes troubling, but not limiting.

### Depth and Breadth

The quotes above demonstrate how the blogs enabled the collection of data that are contextualized within the lives, priorities, and experiences of the bloggers. These data would be difficult to obtain in either focus groups or interviews. Many of the issues brought up by individuals were repeated in subsequent blogs, sometimes enabling a longitudinal perspective in which researchers could gain insight into the dynamics of the situation from the perspective of the young person, and a temporal view of the impact of both having arthritis and the use of medicines.

The following quote illustrates how, in a single blog, many and diverse issues relating to a young person’s experience of living with arthritis may be presented and illustrates the potential for examining the wider context and importance of these issues for the young person. It enables an understanding of health and medicines in the context of wider positive and negative aspects of life:

Arthritis is annoying because you try to put it in the back of your head but it keeps coming, every day you have to think about it because when you want to do something like eg: run but then you realise if you fall over it could be a trip to the hospital which I really don’t like, not because of the staff or anything like that because you are all lovely but it’s just because I have been so many times there and most of the time it has been bad news which makes it a horrible place if you know that when you go there’s a chance you will come home upset and worried about the next step like an operation. I get angry when other people moan about little things like your legs ache, but I’m not saying that I feel sorry for myself all the time it’s just I would swap with  anyone in a heartbeat not to have this, it’s horrible all the not doing stuff other people can do, but in a way I’m lucky that I can walk at all it’s just that I love sports and getting involved but sometimes I can’t do that which is upsetting because I want to do the things I love. It makes me sound ungrateful but I’m not I think my mom and dad are amazing with what they do with me and support me all the time so I am grateful for that but I just get annoyed when people moan because it’s better than not running or even being in this earth today. If I could change myself eg: not having arthritis I don’t know if I would because it has made me a stronger person and realise that not everything's good in life there are bead things on the way and I have faced some of my fears like operations and needles and I’m proud of myself for that because if I didn’t have this condition then I wouldn’t have become brave and I wouldn’t have faced some of the things that I have faced before.

B..a, female, age 13

Overall, the blogs covered a huge variety of issues. The study objectives and the website provided some framework for the topics that bloggers may wish to write about, and this would inevitably impact on the data obtained. Notwithstanding, bloggers did contribute wide-ranging perspectives and experiences. Table 2 provides examples of the range of topics in the blogs.

**Table 2:** Themes and topics of young people’s blogs.

Themes	Examples of topics	Blogs by young people, n
Identity	Self-image	11
	Feeling normal	12
	Limitation in life	12
	Relationships with peers	11
Arthritis	Physical effects	15
	Pain	10
	Psychosocial effects	22
	Beliefs about arthritis	8
Medication	Does it work?	13
	Side-effects and risks	20
	Use of specific (named) medications	19
	Obtaining information	7
Health services	About health providers/professionals	10

### Young Person-Parent Dyads—Comparing Perspectives

This study also provided an opportunity to analyze data across a small number of parent-young person dyads. Data from young people and parents may relate to similar or different experiences and issues. Data may also highlight similar and potentially conflicting perspectives. Qualitative analysis may enable these viewpoints to be examined in the contexts of the differing concerns and priorities of young people and their parents. For instance, this parent-young person dyad felt differently about the school’s involvement:

At school I know they help her either by rearranging the seating plan in the classroom so her neck is ok or by letting her sit out of certain activities if she cannot manage them and doing something else, college however is another big step – feeling a bit scary for her.

chickflick-parent

sometimes school does not understand, like on Tuesday we had assembly and the chairs in the rows are really close together, it hurts my legs to keep them in that position for all that time, mom keeps on telling me to tell them but I feel awkward about it, hopefully college will be different.

pefkosfan, female, age 15

### Sharing the Experience of Living With Arthritis

For the purposes of the research, the blog site was closed. However, contributions that were effectively giving advice to others may reflect the desire of young people for a forum to interact with others with whom they share experiences:

The advice I would give to any young person that has arthritis would be don’t give up the things you love just because you’ve been diagnosed. if you give up on the active things you love then you will start to give up on other things and start using it as an excuse why not to do things.

Spacecadet, female, age 16

Although not intended to have any “therapeutic role”, several participants commented (both via their blogs and to research team members in clinic) that the experience of blogging had been helpful:

well today is the last day of my blog and I think that this has been a great opportunity to talk about my feelings about my arthritis and just general things really :) thank you to everyone who set this up as a really good website. :)

flower123, female, age 14

Another young person and their parent commented that the experience of blogging had been therapeutic during a difficult period of adjustment following their diagnosis.

### Challenges of Using Blogging as a Research Tool

#### Overview

Despite the many benefits for using blogging to engage with young people with arthritis as discussed, there were a number of challenges with conducting research through blogging.

#### Non-Participation

A number of participants who declined to participate in the study gave reasons to the researchers when they were approached. These included not having enough time, not using the Internet very much, being “too lazy”, busy with studying for their exams, not liking to write, and the perception of a parent that a young person would not wish to participate.

Some other reasons that were not explicitly stated, but hypothesized by the research team, include not knowing what to write on a blog, preference for outdoor rather than indoor activities, not wanting to think about their illness when they were well, and problems in accessing the site at home, for example, due to permissions that are required to run the website.

The specific reasons given by the young people may indicate how blogging in research of this kind may not be suitable for all young people.

#### Not Knowing What to Write

Some young people indicated to the research team that they did not know what to write about and were unsure as to whether what they were writing about would be deemed important or significant. The prompts posted by the research team on possible issues to blog about were intended to reduce this anxiety. Perhaps more reference should have been made to these prompts throughout the study, but the research team wanted to avoid “telling” the young people what to write about, as the goal of the project was to find out about was important to them.

#### Technical Problems

There were also some technical issues. Access to the Arthriting site was restricted by schools and workplaces with firewalls, although participants would have been able to access the site from home or through a smartphone. Access was actually restricted in the pediatric hospital itself, which reduced opportunities to demonstrate the site during the informed consent procedures.

## Discussion

### Principal Findings

This project offered a novel way of engaging young people in research, by creating a secure individual blog-space. Young people blogged to different extents; not all took up the blogging opportunity, but others enjoyed the experience and their blogs provided valuable insights relevant to our study objectives.

### Strengths and Limitations

This methodology did present a number of limitations. First, the sample was self-selecting. Despite all eligible young people being invited to take part, many declined. The sample may reflect only those who feel competent with Web-based activities and who have reasonable literacy skills. We likely missed out on the insights of young people who do not consider Web-based resources relevant to their own condition. A recent study emphasized that not all young people choose to seek information about pain online [30]. Some of the reasons given for non-participation in our study indicate that the methodology may not suit all; this approach might be most effective if combined with other inclusive qualitative methods, such as telephone interviewing. This might promote involvement of a more representative sample. We saw substantial attrition at each recruitment stage of the study, and the expectation of a 2-month engagement might have been too ambitious.

The blogs (ie, the dataset) may also not be fully reflective of experiences and perspectives of the participants. This methodology would not necessarily be expected to provide a systematic and comprehensive dataset. For example, it could be that the young people were more likely to blog when they had problems or issues with their arthritis that made them focus on their condition and perhaps blog more. The blogs, often rich in context, need to be analyzed with this in mind, with each data item providing a perspective important to a young person at a particular point in time and in the context of other aspects of their life and priorities. The data enabled “how” and “why” questions to be explored in the analysis. When discussing the viability and relative strengths of blogging as a research methodology, it is necessary also to consider issues of reliability and validity. If we were to compare this with interview and focus group methodologies, then this method had no interviewer or transcriber bias. In the use of “audio-diaries”, Sargeant and Gross [16] similarly described how they achieved “a view of how the disease fitted into individual lives”.

In line with principles of qualitative inquiry, the data collection was “participant-led”. Young people were in control of their involvement. They determined what they wrote about, how much they wrote, and how often they visited the site. When young people expressed difficulty regarding what they should blog about, the research team—while providing appropriate assistance (possible categories on the website)—wished to avoid influencing their blogs.

In our quest for security, the blogs were for private use by each individual and were not associated with a community where experiences and issues could be shared. This might also have been off-putting to those who wished to communicate with others. Conversely, others may have been willing to disclose feelings and experiences that they may have been reluctant to share in a Web-based community setting. The desire of some young people with a less common condition, like juvenile arthritis, to “meet” and compare experiences could be addressed by a dedicated Web-based forum. Although a young person’s discussion forum does exist for young people with arthritis, this can be dominated by older adolescents and adults and is thus not appealing to the age group of “younger young people” that we engaged.

### Conclusions

Web-based research with young people presents opportunities and challenges for researchers. Web-based blogging methodology has the potential to give young people and parents the space and empowerment to express their own ideas and concerns. However, this project suggests that it might not be the best way to engage a large diverse group of young people and might most effectively be combined with other approaches. Despite this, the study provided valuable data on the experience and impact of living with a long-term condition from the perspectives of young people with arthritis.

## Abbreviations

IBD: inflammatory bowel disease
JIA: juvenile idiopathic arthritis
PPRT: Pharmacy Practice Research Trust
PTECO: Pharmaceutical Trust for Educational and Charitable Objects
